# Supplementary material for: Phenotypic Diversity of Lactobacillus casei Group Isolates as a Selection Criterion for Use as Secondary Adjunct Starters
Source: Microorganisms. 2020 Jan 17;8(1):128. doi: 10.3390/microorganisms8010128 (PMC7022476; doi:10.3390/microorganisms8010128)
Supplement: Supplementary file 1 [file microorganisms-08-00128-s001.zip › Supplementary Table 2.docx]

**Supplementary Table 2:** Accession numbers of the publicly available *L. casei* group nucleotide sequences used for degenerate primers design.

| Strain | Accession number |
| --- | --- |
| *L. paracasei* ATCC 334 | NC_008526.1 |
| *L. paracasei* ssp. *paracasei* JCM 8130 | AP012541.1 |
| *L. paracasei* ssp. *paracasei* 8700.2 | CP002391.1 |
| *L. casei* 12A | NZ_CP006690 |
| *L. casei* LOCK919 | NC_021721 |
| *L. casei* W56 | NC_018641 |
| *L. casei* BD-II | NC_017474 |
| *L. casei* LC2W | CP006690.1 |
| *L. casei* str. Zhang | NC_014334.2 |
| *L. casei* BL23 | NC_017473 |
| *L. rhamnosus* LOCK908 | CP005485.1 |
| *L. rhamnosus* LOCK900 | NC_021723.1 |
| *L. rhamnosus* ATCC 8530 | NC_017491.1 |
| *L. rhamnosus* LC 705 | NC_013199.1 |
| *L. rhamnosus* GG | NZ_CP031290.1 |
| *L. rhamnosus* GG (ATCC 53103) | NC_013198.1 |
