# Supplementary material for: Phenotypic Diversity of Lactobacillus casei Group Isolates as a Selection Criterion for Use as Secondary Adjunct Starters
Source: Microorganisms. 2020 Jan 17;8(1):128. doi: 10.3390/microorganisms8010128 (PMC7022476; doi:10.3390/microorganisms8010128)
Supplement: Supplementary file 1 [file microorganisms-08-00128-s001.zip › Supplementary Figure 1.docx]

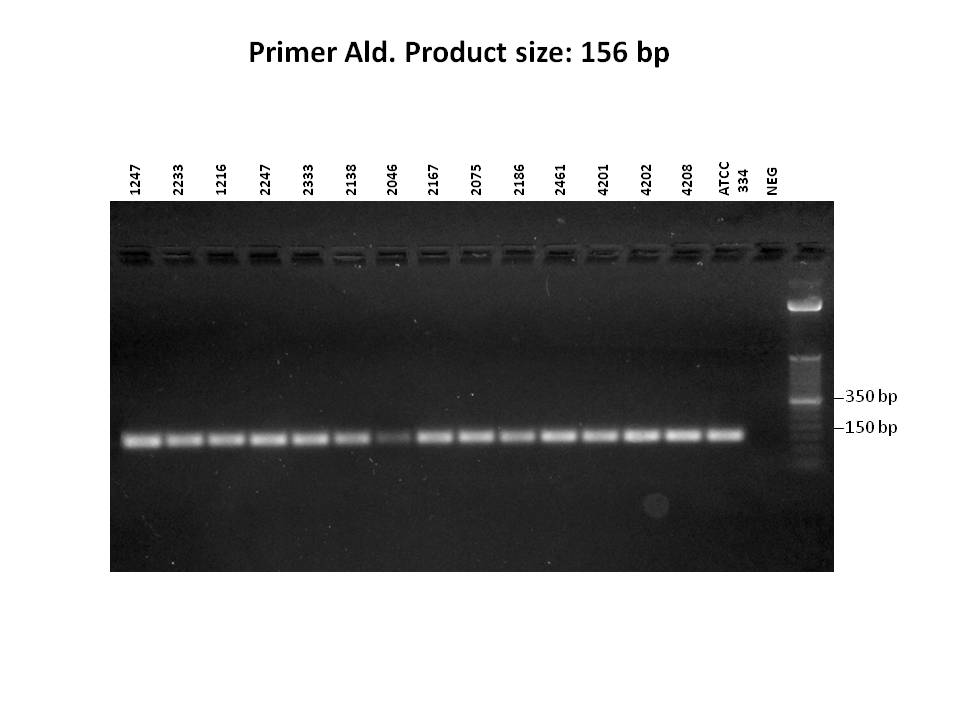

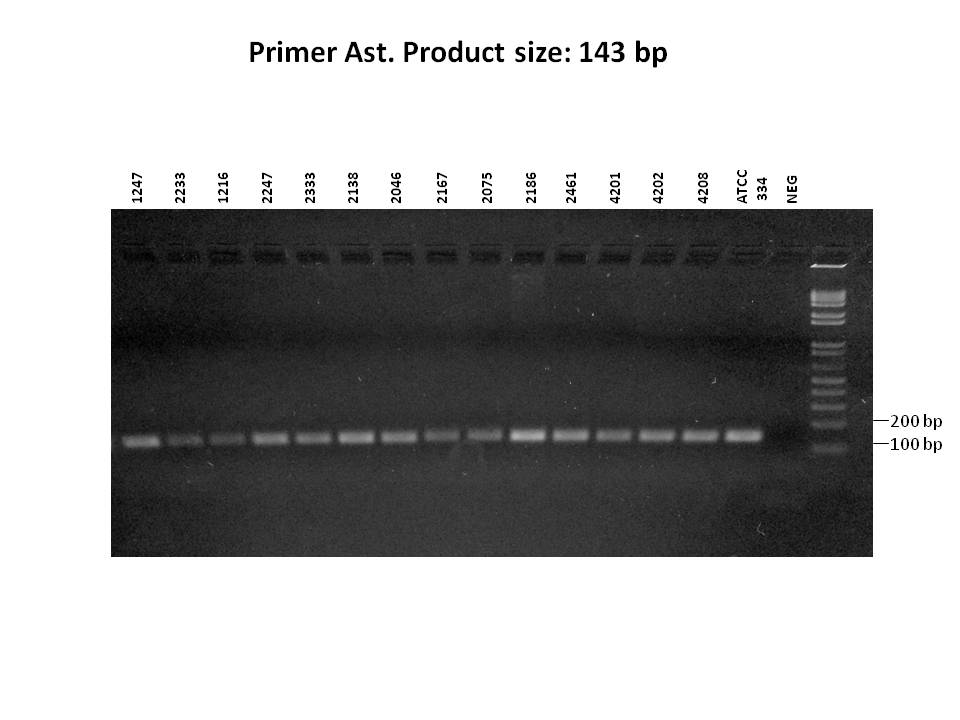


**(c)**

**(b)**

**(a)**

**
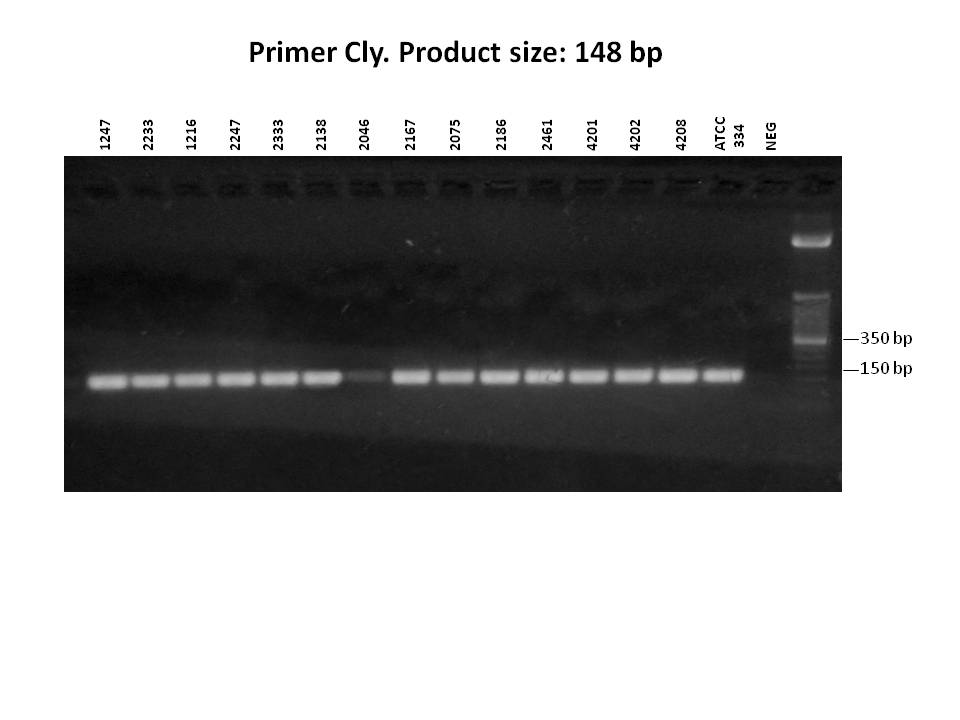
**

**Supplementary Figure 1:** End-point PCR with degenerate primers on the selected *L. casei* isolates. **(a)** Ald primers, product size: 156 bp, Marker: 50 bp DNA Ladder (Invitrogen); **(b)** Ast primers, product size: 143 bp, Marker: 1 Kb plus DNA Ladder (Invitrogen); **(c)** Cly primers, product size: 141 bp, Marker: 50 bp DNA Ladder (Invitrogen).
