# Supplementary material for: Phenotypic Diversity of Lactobacillus casei Group Isolates as a Selection Criterion for Use as Secondary Adjunct Starters
Source: Microorganisms. 2020 Jan 17;8(1):128. doi: 10.3390/microorganisms8010128 (PMC7022476; doi:10.3390/microorganisms8010128)
Supplement: Supplementary file 1 [file microorganisms-08-00128-s001.zip › Supplementary Table 1.docx]

**Supplementary Table 1:** Concentration of volatile compounds measured milk fermented with the selected strains determined by GC-MS-SPME. Data are expressed as mg/L, the reported values are the mean ± standard deviation (n=2).

|  |  | ***L. casei*** | | | ***L. paracasei*** | | | | | | | ***L. rhamnosus*** | | | |
| --- | --- | --- | --- | --- | --- | --- | --- | --- | --- | --- | --- | --- | --- | --- | --- |
| **Compounds** | **Abbr.** | **1247** | **2046** | **2138** | **2247** | **2333** | **2186** | **2461** | **4201** | **4202** | **4208** | **2233** | **1216** | **2167** | **2075** |
| acetic acid | **AC1** | 55.2 ± 13.1 | 166.2 ± 47.1 | 206.7 ± 57.3 | 242.2 ± 109.5 | 67.3 ± 43.3 | 107 ± 60 | 122.3 ± 59.8 | 60.9 ± 30.8 | 157.9 ± 73.4 | 139.4 ± 40.9 | 87.9 ± 7.2 | 118.2 ± 43.1 | 163 ± 45.4 | 292.1 ± 83.8 |
| 2-methyl-propanoic acid | **AC2** | 0.1 ± 0 | 0.5 ± 0.4 | 0.5 ± 0.3 | 0.6 ± 0.6 | 0.3 ± 0.2 | 0.1 ± 0.1 | 0.7 ± 0.6 | 0.5 ± 0.4 | 0.5 ± 0.4 | 0.5 ± 0.4 | 0.1 ± 0 | 0.2 ± 0.1 | 0.5 ± 0.4 | 0.5 ± 0.4 |
| butanoic acid | **AC3** | 9 ± 1.4 | 13.8 ± 3.2 | 14.7 ± 4.5 | 20.2 ± 8.9 | 16.5 ± 9.1 | 10.2 ± 5.9 | 14.8 ± 6 | 15.6 ± 6.8 | 13.4 ± 5 | 16.8 ± 5.3 | 11.2 ± 0.6 | 15.8 ± 3.5 | 17.1 ± 4.9 | 20.3 ± 7.4 |
| 2-methyl-hexanoic acid | **AC4** | 1.4 ± 0.2 | 2.1 ± 0.7 | 2.4 ± 0.7 | 3.4 ± 1.5 | 2.9 ± 1.5 | 0.8 ± 0.1 | 2.4 ± 1.2 | 2.7 ± 1.4 | 2.5 ± 1.1 | 2.7 ± 0.8 | 1.6 ± 0.1 | 1.4 ± 0.5 | 2.7 ± 0.8 | 3.2 ± 1.2 |
| 3-hydroxy-decanoic acid | **AC5** | 0 ± 0 | 0.2 ± 0.2 | 0.2 ± 0.2 | 0.3 ± 0.3 | 2.6 ± 2.6 | 0.2 ± 0.1 | 0.3 ± 0.3 | 0.2 ± 0.1 | 0.3 ± 0.3 | 0.1 ± 0.1 | 0 ± 0 | 0.2 ± 0.2 | 0.2 ± 0.2 | 0.3 ± 0.2 |
| hexanoic acid | **AC6** | 14.2 ± 2.9 | 22 ± 5.7 | 30.3 ± 10.2 | 40 ± 20.9 | 37.7 ± 23.4 | 23.2 ± 11.6 | 16.7 ± 7.3 | 27.2 ± 16.7 | 19 ± 9 | 29.2 ± 9.7 | 18.7 ± 0.8 | 30.2 ± 11.7 | 33.8 ± 15.9 | 38 ± 16.3 |
| octanoic acid | **AC7** | 1.7 ± 1.5 | 0.9 ± 0.9 | 7.2 ± 1.1 | 9.8 ± 6.2 | 8.8 ± 8.6 | 2.7 ± 2.6 | 0 ± 0 | 4.5 ± 4.5 | 0 ± 0 | 8.4 ± 4.1 | 5.9 ± 0.3 | 9.7 ± 5.1 | 8.7 ± 6.2 | 9.4 ± 5.1 |
| benzoic acid | **AC8** | 0.4 ± 0.3 | 0.1 ± 0.1 | 0.2 ± 0.2 | 0.7 ± 0.2 | 1.5 ± 1.4 | 0 ± 0 | 0 ± 0 | 1.1 ± 1 | 10.7 ± 10.3 | 1.7 ± 1.6 | 1.7 ± 0.1 | 4.7 ± 3.5 | 0.4 ± 0.4 | 1.5 ± 1.4 |
| **Acids** |  | 82.2 ± 19.5 | 205.8 ± 58.2 | 262.3 ± 74.7 | 317.3 ± 148.1 | 137.7 ± 90.3 | 144.1 ± 80.6 | 157.4 ± 75.3 | 112.7 ± 61.8 | 204.4 ± 99.4 | 198.7 ± 63 | 127.1 ± 8.9 | 180.3 ± 67.7 | 226.4 ± 74.2 | 365.4 ± 116 |
| 2-octanol | **AL1** | 0.2 ± 0.1 | 1.1 ± 0.1 | 3.4 ± 1.5 | 1.5 ± 0.6 | 1.6 ± 1.1 | 0.7 ± 0.5 | 0.1 ± 0.1 | 0.8 ± 0.4 | 0.8 ± 0.2 | 0.6 ± 0.2 | 3.1 ± 0.4 | 2.8 ± 1 | 2.2 ± 0.2 | 6.4 ± 1.6 |
| 3-methyl-3-buten-1-ol | **AL2** | 1.3 ± 0.2 | 2.2 ± 0.3 | 3.7 ± 0.7 | 4.9 ± 0.9 | 6.1 ± 1.1 | 1.8 ± 0.2 | 3.1 ± 0.9 | 6.2 ± 1 | 2.6 ± 0.9 | 7.2 ± 1.2 | 3.7 ± 0.1 | 3.9 ± 0.6 | 3 ± 0.7 | 3.4 ± 1 |
| 2-heptanol | **AL3** | 0.2 ± 0.1 | 0.2 ± 0 | 0.3 ± 0.2 | 0.6 ± 0.3 | 0.3 ± 0 | 0.2 ± 0.1 | 0.4 ± 0.2 | 0.5 ± 0.2 | 0.8 ± 0.3 | 0.5 ± 0.2 | 0.1 ± 0 | 0.1 ± 0 | 0.1 ± 0.1 | 0.1 ± 0 |
| 3-methyl-2-buten-1-ol | **AL4** | 0.8 ± 0.1 | 1.5 ± 0.6 | 2.2 ± 1.7 | 2.7 ± 2.1 | 3.8 ± 1.3 | 0.9 ± 0.4 | 1.5 ± 1.7 | 2.9 ± 2.9 | 1.3 ± 1.1 | 3.1 ± 3.4 | 1.7 ± 0.1 | 2.3 ± 1.9 | 3.2 ± 0.9 | 3.3 ± 1.3 |
| 1-hexanol | **AL5** | 0.3 ± 0 | 1.6 ± 0.5 | 8.7 ± 4 | 0.6 ± 0.2 | 0.8 ± 0.5 | 0.6 ± 0.4 | 0.4 ± 0.2 | 0.6 ± 0.1 | 0.4 ± 0.1 | 0.5 ± 0.2 | 0.5 ± 0.1 | 2.3 ± 0.4 | 3.6 ± 1 | 5.4 ± 0.9 |
| 2-ethyl-1-hexanol | **AL6** | 1 ± 0.1 | 1.4 ± 0.4 | 1.5 ± 0.3 | 1.9 ± 0.8 | 3.1 ± 2.1 | 1.1 ± 0.6 | 1.8 ± 0.8 | 1.8 ± 0.8 | 1.8 ± 0.5 | 1.4 ± 0.5 | 0.7 ± 0.1 | 0.9 ± 0.2 | 1.5 ± 0.5 | 2 ± 0.7 |
| 2-furanmethanol | **AL7** | 0.5 ± 0 | 0.7 ± 0.3 | 0.9 ± 0.4 | 1.1 ± 0.6 | 1.2 ± 0.7 | 0.4 ± 0.2 | 0.9 ± 0.4 | 1.1 ± 0.5 | 0.9 ± 0.4 | 0.8 ± 0.2 | 0.4 ± 0 | 0.3 ± 0.1 | 0.9 ± 0.4 | 0.9 ± 0.3 |
| **Alcohols** |  | 4.3 ± 0.6 | 8.7 ± 2.3 | 20.7 ± 8.7 | 13.3 ± 5.5 | 17 ± 6.8 | 5.8 ± 2.4 | 8.2 ± 4.3 | 14 ± 5.8 | 8.6 ± 3.6 | 14.1 ± 6 | 10.2 ± 0.8 | 12.6 ± 4.3 | 14.5 ± 3.8 | 21.5 ± 5.8 |
| octanoic acid ethyl ester | **ES1** | 0.7 ± 0 | 3.4 ± 2.8 | 2.4 ± 1.7 | 3.8 ± 3.2 | 2.1 ± 1.5 | 2 ± 1.3 | 6.1 ± 5.6 | 1.1 ± 0.5 | 4.7 ± 4.2 | 1 ± 0.5 | 0.5 ± 0 | 2.3 ± 1.7 | 3.4 ± 2.8 | 2.4 ± 1.7 |
| decanoic acid ethyl ester | **ES2** | 0.2 ± 0 | 1.5 ± 1.4 | 1.3 ± 1.2 | 1.9 ± 1.8 | 1.2 ± 1.2 | 1.2 ± 1 | 2.4 ± 2.3 | 1 ± 0.8 | 2.2 ± 2.1 | 0.4 ± 0.2 | 0 ± 0 | 0.9 ± 0.8 | 1.5 ± 1.4 | 1.7 ± 1.6 |
| hexanoic acid ethyl ester | **ES3** | 0 ± 0 | 0.2 ± 0.2 | 0.1 ± 0.1 | 0.1 ± 0.1 | 0.1 ± 0.1 | 0.1 ± 0.1 | 0.3 ± 0.2 | 0 ± 0 | 0.2 ± 0.2 | 0 ± 0 | 0 ± 0 | 0.1 ± 0.1 | 0.1 ± 0.1 | 0.1 ± 0.1 |
| **Esters** |  | 0.9 ± 0.1 | 5 ± 4.4 | 3.7 ± 3 | 5.8 ± 5.1 | 3.4 ± 2.8 | 3.4 ± 2.4 | 8.8 ± 8.1 | 2.1 ± 1.3 | 7.1 ± 6.5 | 1.4 ± 0.8 | 0.6 ± 0 | 3.3 ± 2.6 | 4.9 ± 4.3 | 4.3 ± 3.4 |
| 2-propanone | **K1** | 2 ± 0.3 | 2.2 ± 0.2 | 2.1 ± 0.9 | 3 ± 0.8 | 0.6 ± 0.3 | 2.7 ± 1.9 | 4.1 ± 0.4 | 4.2 ± 0.7 | 2.9 ± 1 | 3.2 ± 0.8 | 2.5 ± 0.1 | 1.4 ± 0.9 | 1.4 ± 1 | 3.1 ± 0.4 |
| 6,10-dimethyl-5,9-undecadien-2-one | **K2** | 5.4 ± 0 | 4.7 ± 0.9 | 5 ± 2 | 8.7 ± 3.2 | 2.9 ± 1.9 | 3.4 ± 2.6 | 8.7 ± 3.5 | 8.7 ± 2.5 | 7 ± 1.4 | 7 ± 2.3 | 4.9 ± 0.5 | 3.5 ± 2.4 | 5.6 ± 0.7 | 6.5 ± 1.4 |
| 2-pentanone | **K3** | 3.4 ± 0.3 | 3.5 ± 0.6 | 3.2 ± 1.1 | 3.5 ± 0.9 | 1.9 ± 1.6 | 3.4 ± 2.1 | 4.7 ± 1.5 | 5.5 ± 1.5 | 3.7 ± 0.5 | 4.1 ± 0.9 | 4 ± 0 | 2.7 ± 1 | 4.5 ± 0.8 | 3.5 ± 1.3 |
| diacetyl | **K4** | 1.5 ± 0.3 | 10.9 ± 4 | 1.1 ± 0.1 | 8.3 ± 1 | 1.1 ± 0 | 10 ± 0.8 | 8.6 ± 1.3 | 1.8 ± 0.1 | 2.5 ± 1 | 3.1 ± 0.5 | 4.8 ± 0.4 | 5.9 ± 4.1 | 1.8 ± 0.3 | 4.3 ± 1.6 |
| 2-heptanone | **K5** | 37.2 ± 1.2 | 59.7 ± 14.4 | 58.5 ± 21.6 | 73.7 ± 27.9 | 30.4 ± 17.3 | 46.5 ± 22.3 | 88.1 ± 42.4 | 81.8 ± 22 | 69.6 ± 16 | 64.4 ± 20.4 | 38.6 ± 7.3 | 41 ± 6.6 | 70.2 ± 21.9 | 70.8 ± 13 |
| 2-octanone | **K6** | 0.1 ± 0 | 0.2 ± 0.1 | 0.2 ± 0.1 | 0.2 ± 0.1 | 0.1 ± 0 | 0.1 ± 0.1 | 0.3 ± 0.1 | 0.3 ± 0.1 | 0.3 ± 0.1 | 0.2 ± 0 | 0.1 ± 0 | 0.1 ± 0 | 0.2 ± 0.1 | 0.2 ± 0.1 |
| acetoin | **K7** | 11.1 ± 0.9 | 119.2 ± 28.1 | 168.2 ± 47.6 | 125.4 ± 46.8 | 5.9 ± 2.5 | 76 ± 19 | 41.1 ± 17.4 | 9.9 ± 2.4 | 49.8 ± 11 | 37.5 ± 9.3 | 36.8 ± 0.6 | 77.6 ± 13.9 | 91.8 ± 14.1 | 114.9 ± 27.6 |
| 4-sec-butoxy-2-butanone | **K8** | 0 ± 0 | 0.3 ± 0 | 0.2 ± 0 | 0.3 ± 0.1 | 2.5 ± 2.2 | 0.9 ± 0.6 | 0.3 ± 0.2 | 0.4 ± 0.1 | 0.4 ± 0.2 | 0.4 ± 0.3 | 0.1 ± 0 | 0.5 ± 0.4 | 0.4 ± 0.1 | 1.3 ± 0.9 |
| 1-hydroxy-2-propanone | **K9** | 0.8 ± 0.2 | 0.2 ± 0 | 0.6 ± 0.2 | 2 ± 0.8 | 0.4 ± 0.4 | 0.2 ± 0.1 | 1.1 ± 0.5 | 1.7 ± 0.7 | 1.1 ± 0.5 | 2.5 ± 0.9 | 0.5 ± 0.1 | 0.4 ± 0.1 | 1.7 ± 0.4 | 0.2 ± 0.2 |
| 2-nonanone | **K10** | 5.5 ± 0.5 | 9.1 ± 2.4 | 9.1 ± 3.3 | 10.8 ± 3.8 | 5.6 ± 2.6 | 6.7 ± 1.8 | 10.3 ± 3.7 | 11.2 ± 2.8 | 8.9 ± 3.9 | 10 ± 3.5 | 6.7 ± 0.3 | 6 ± 0.5 | 12.1 ± 3.3 | 11.7 ± 4 |
| 2-undecanone | **K11** | 1.1 ± 0.1 | 1.8 ± 0.5 | 2.1 ± 0.8 | 2.5 ± 1.1 | 2.2 ± 0.7 | 1.6 ± 0.3 | 2.2 ± 0.7 | 2.3 ± 0.8 | 2.3 ± 0.7 | 1 ± 0.5 | 1.1 ± 0 | 1.6 ± 0.2 | 2.2 ± 0.8 | 2.8 ± 1 |
| **Ketones** |  | 68.2 ± 3.9 | 211.7 ± 51.2 | 250.4 ± 77.6 | 238.5 ± 86.4 | 53.8 ± 29.6 | 151.5 ± 51.5 | 169.5 ± 71.7 | 127.9 ± 33.5 | 148.5 ± 36.1 | 133.3 ± 39.4 | 100.3 ± 9.5 | 140.7 ± 29.9 | 192.1 ± 43.5 | 219.4 ± 51.6 |
| **2,4-dimethyl-1-eptene** | **O1** | 6 ± 1.2 | 5.4 ± 2 | 7.9 ± 2.9 | 4.6 ± 0.2 | 3.8 ± 3.6 | 11 ± 2.1 | 7.7 ± 1.6 | 7.4 ± 0.8 | 7.2 ± 0.2 | 3 ± 1.2 | 7.2 ± 1.5 | 7.9 ± 2 | 3.6 ± 0.7 | 8.3 ± 2.9 |
| **5-ethyl-2,2,3-trimethyl-heptane** | **O2** | 2.2 ± 0.2 | 2.9 ± 0.8 | 2.9 ± 0.8 | 3.6 ± 0.8 | 3 ± 0.4 | 2.8 ± 0.5 | 3 ± 0.8 | 2.7 ± 0.9 | 2.2 ± 0.2 | 2.3 ± 0.1 | 2.8 ± 0 | 2.6 ± 0.1 | 2.7 ± 0.6 | 3 ± 0.9 |
| **3-hydroxy butanal** | **O3** | 0.5 ± 0.1 | 0.4 ± 0.2 | 0.4 ± 0.2 | 0.4 ± 0.3 | 0.4 ± 0.1 | 0.3 ± 0 | 0.4 ± 0.2 | 0.3 ± 0.2 | 0.3 ± 0.2 | 0.4 ± 0.3 | 0.6 ± 0 | 0.5 ± 0.1 | 0.4 ± 0.3 | 0.5 ± 0.3 |
| **Others** |  | 8.7 ± 1.5 | 8.8 ± 2.9 | 11.2 ± 4 | 8.6 ± 1.2 | 7.2 ± 4.1 | 14.1 ± 2.7 | 11 ± 2.6 | 10.4 ± 1.9 | 9.7 ± 0.5 | 5.7 ± 1.6 | 10.6 ± 1.5 | 11 ± 2.3 | 6.7 ± 1.6 | 11.8 ± 4 |
